# Thermal Insulation Mechanism, Preparation, and Modification of Nanocellulose Aerogels: A Review

**DOI:** 10.3390/molecules28155836

**Published:** 2023-08-03

**Authors:** Yueqi Wu, Xue Wang, Lihong Yao, Siyu Chang, Ximing Wang

**Affiliations:** College of Materials Science and Art Design, Wood Science and Technology, Inner Mongolia Agricultural University, Hohhot 010018, China; wxx@emails.imau.edu.cn (Y.W.); wangxue@imau.edu.cn (X.W.); csy792111@emails.imau.edu.cn (S.C.); w_ximing@263.net (X.W.)

**Keywords:** nanocellulose, aerogel, thermal insulation, mechanical properties, flame retardance, hydrophobicity

## Abstract

Energy problems have become increasingly prominent. The use of thermal insulation materials is an effective measure to save energy. As an efficient energy-saving material, nanocellulose aerogels have broad application prospects. However, nanocellulose aerogels have problems such as poor mechanical properties, high flammability, and they easily absorbs water from the environment. These defects restrict their thermal insulation performance and severely limit their application. This review analyzes the thermal insulation mechanism of nanocellulose aerogels and summarizes the methods of preparing them from biomass raw materials. In addition, aiming at the inherent defects of nanocellulose aerogels, this review focuses on the methods used to improve their mechanical properties, flame retardancy, and hydrophobicity in order to prepare high-performance thermal insulation materials in line with the concept of sustainable development, thereby promoting energy conservation, rational use, and expanding the application of nanocellulose aerogels.

## 1. Introduction

Global energy consumption is gradually increasing. Developing new energy-efficient materials is urgently needed. Using thermal insulation materials is effective in energy conservation, especially in the case of the energy shortage and low utilization rates [1]. Common commercial thermal insulation materials such as mineral wool and polymer foam are made of non-renewable resources, with limitations such as environmental unfriendliness, non-biodegradability, and poor mechanical stability [2]. Lightweight, high-performance, multi-functional composite, and environmentally friendly thermal insulation materials are required. Nanoporous thermal insulation materials enable a more than 50% reduction in insulation thickness according to the passive house standard compared with commercial EPS [3]. Their thermal conductivity is sufficiently low to make them very promising.

Preparing aerogels based on biodegradable polysaccharides alleviates the crisis of non-renewable resource shortages and environmental pollution and has excellent research value and development prospects. The dissolution of raw materials and the formation of gels are critical aspects and challenges in preparing polysaccharide-based aerogels. Compared to polysaccharide materials like chitosan, chitin, and pectin, nanocellulose exhibits good solubility and can evenly disperse to form a gel in aqueous solution [4]. In addition, nanocellulose obtained from green biomass has a high aspect ratio and meshed twining structure. The nanocellulose suspension forms a stable three-dimensional network structure due to hydrogen bonds and entanglement between the fibrils; upon solvent removal using a particular drying method, ultralight aerogels with high porosity and low thermal conductivity can be produced [5]. Studies on nanocellulose-based aerogels with superthermal insulation and superlight properties have been reported in recent years [6,7,8]. The gas–solid two-phase interpenetrated nano-framework of nanocellulose aerogels is interconnected to form a mesoporous structure. Their thermal conductivity can be as low as 0.025 W m^−1^K^−1^, which surpasses commercially dominant materials like expanded polystyrene, polyurethane foam, and glass wool in terms of thermal insulation performance. Thus, nanocellulose aerogels are expected to be used as a potential high-performance thermal insulation materials [9,10]. With the development of more environmentally friendly and industrially feasible nanocellulose modification and processing methods, as well as the exploration of large-scale drying methods, the production costs of nanocellulose aerogels will be significantly lower than those of commercial thermal insulation materials with the best thermal insulation performance, such as vacuum insulation and silica aerogel [11]. The surface of nanocellulose is rich in hydroxyl groups, which is advantageous in various chemical modifications to achieve high performance and a multi-functional complex. Compared with traditional and organic aerogels, nanocellulose aerogels possess a range of unique properties, such as flexibility, degradability, and biocompatibility. Using nanocellulose to prepare renewable thermal insulation materials has great potential and it is widely applied in building materials [12], electronic equipment [13], the clothing industry [14], etc. 

**Figure 1:**
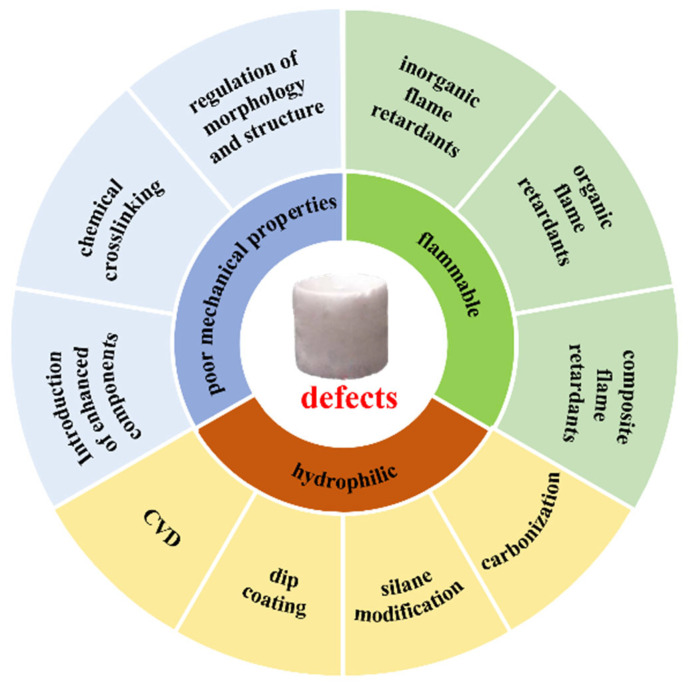
Problems of nanocellulose aerogels and modification strategies.

However, the practical applications of nanocellulose aerogels face many challenges, as shown in Figure 1. Hydrogen bonds and physical crosslinking between cellulose molecules mainly connect their internal structure. Their mechanical strength, especially their compression resilience, is poor [15,16]. Nanocellulose aerogels are inevitably squeezed during storage and use. Changes in the internal structure after removing the external force cause profound changes in their physical properties, including porosity, specific surface area, and thermal conductivity. Nanocellulose is a hydrophilic biomass polymer. Due to the abundant hydroxyl groups on the surface, pure nanocellulose aerogels effortlessly absorb water in the environment. The high thermal conductivity of water enhances the internal heat transfer of the materials and ultimately reduces the thermal insulation effect, limiting their application [17]. Cellulose, mainly composed of C, H, and O elements, is flammable [18,19]. Nanocellulose aerogels prepared from cellulose do not melt at high temperatures. But when exposed to open flame, the flame spreads quickly, burning rapidly and generating a large amount of smoke, significantly reducing the effectiveness and safety of nanocellulose aerogels.

This review article, therefore, analyzes the thermal insulation mechanism of nanocellulose aerogels and summarizes the methods of preparing them from biomass raw materials. In addition, aiming at the inherent defects of nanocellulose aerogels, this review summarizes the methods used to improve their mechanical properties, flame retardancy, and hydrophobicity in order to prepare high-performance thermal insulation materials. Using them in construction, warehousing, packaging, clothing insulation, and power batteries for new energy vehicles provides high value-added technical routes for utilizing biomass resources and promotes energy conservation and rational utilization.

## 2. Thermal Insulation Mechanism of Nanocellulose Aerogels

### 2.1. Insulation Mechanism

**Figure 2:**
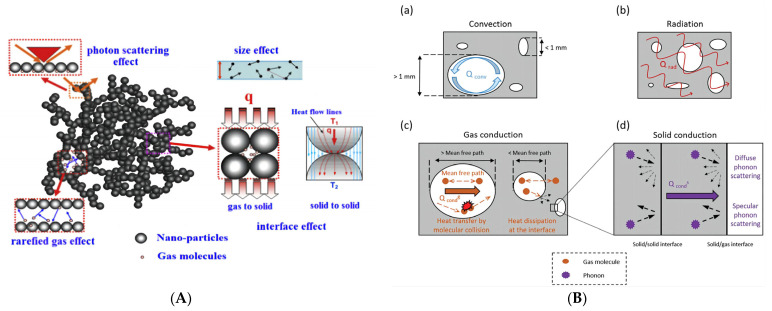
Heat transfer mechanism of nanocellulose aerogels. (**A**) Common effects on heat transfer in nanoporous insulation materials [17]. (**B**) The modes of heat transport in porous materials [10].

Nanocellulose aerogels are solid materials with low density, high porosity, and a controllable structure. As shown in Figure 2, the heat transfer mechanism of porous materials is usually classified as convection (*λ_conv_*), gas conduction (*λ_gas_*), solid conduction (*λ_solid_*), and radiation (*λ_rad_*). The total effective thermal conductivity is expressed as [20]:

(1) $$\lambda_{tot}=\lambda_{conv}+\lambda_{gas}+\lambda_{solid}{+\lambda }_{rad}$$

Convection refers to the heat transfer through gas using the temperature gradient inside the material. The contribution from convection is negligible when the pore size of the nanocellulose aerogel is less than 1 mm [21,22].

Gas conduction refers to the heat transfer through gas molecule collision. The thermal conductivity of gas conduction can be calculated using the following formulas [23,24]:

(2) $$\lambda_{gas}=\frac{\lambda_{g0}}{1+2\beta K_{n}}$$

(3) $$K_{n}=\frac{l_{mean}}{\varphi }$$

(4) $$l_{\mathrm{mean}}=\frac{K_{B}T}{\sqrt{2}\sigma P_{g}}$$

where *λ_g_*_0_ is the thermal conductivity of the gas in free motion; *β* is the constant of energy transfer effectiveness between gas molecules and solid pore walls, and its value is 1.5–2.0; *K_n_* is the Knudsen number; *l_mean_* is the mean free path; *φ* can be understood as the pore diameter of the material; *K_B_* denotes the Boltzmann constant, and its value is 1.38 × 10^−23^ J/K; *T* represents the temperature; *σ* is the cross-sectional area of the gas molecule, and its value is is about 0.4 nm^2^ for nitrogen and oxygen, as the main constituents of air; and *P_g_* refers to the gas pressure. From the above equations, it can be seen that gas conduction depends on the pore size and gas molecular mean free path in the porous structure, and is also affected by the density and specific surface area. The gaseous thermal conductivity in large pores is higher than that in small pores. The number of large pores falls as the uniformity of pore size distribution increases, reducing the heat transfer by gas molecules in nanoporous materials.

Solid conduction mainly depends on the lattice vibration of the solid molecule around its equilibrium position. A phonon is defined as the minimum energy required for the lattice vibration of the molecule in a solid. According to the kinetic theory formula, the following equation determines the thermal conductivity of solid conduction [17,25]:

(5) $$\lambda_{s}=\frac{1}{3}\rho c_{v}v_{\alpha }\Lambda_{ph}=\frac{1}{3}\rho c_{v}{v_{\alpha }}^{2}\tau_{ph}$$

where *ρ* indicates the density; *c_v_* is the crystal-specific heat capacity, which can be obtained from the Deybe model; *v_α_* represents the average velocity of the phonon; Λ*_ph_* is the phonon mean free path; and *τ_ph_* is the phonon relaxation time. For porous insulation materials, solid conduction can be significantly reduced by lessening the characteristic size of the solid phase framework to make it close to the phonon mean free path [26]. Decreasing the density can reduce the solid conduction per square meter. Constructing a three-dimensional porous structure can make the heat transfer route more complicated and extend the heat transfer path through the solid, thereby significantly reducing solid conduction [25,27].

Radiation refers to the heat transfer of an object with a temperature above absolute zero through infrared radiation. At normal temperature and pressure, radiation conduction is negligible, but when the thermal conductivity and density of porous materials are low, radiation conduction needs to be considered [28]. The thermal conductivity of radiation is usually expressed as [29,30]:

(6) $$\lambda_{rad}=\frac{16n^{2}\sigma_{B}}{3\rho K_{e,m}}T^{3}$$

where *n* is the refractive index (the refractive index of aerogels is approximately 1); $\sigma_{B}$ is the Stefan-Boltzmann constant, and its value is 5.67 × 10^−8^ W/(m^2^·K^4^); ρ is the density of the insulation material; $K_{e,m}$ is the mean extinction coefficient; and T is the temperature. It is apparent that radiation conduction is related to temperature, material density, and the extinction coefficient. The multi-level ordered microstructure creates an “infinite shielding effect” in nanocellulose aerogels, effectively decreasing radiation heat transfer [31].

In summary, the thermal insulation performance of nanocellulose aerogels is mainly influenced by their pore size, pore size distribution, density, characteristic size of the solid phase framework, and microstructure. When the pore size is smaller than the mean free path of gas molecules, the gas molecule free movement is severely restricted, thus decreasing the value of λ_gas_. The more uniform the pore size distribution, the fewer large pores, and the smaller the value of λ_gas_. As the density is reduced, the porosity increases, and the solid conduction per square meter decreases, resulting in a diminution of λ_gas_ and λ_s_. When the characteristic size of the solid phase framework is similar to the mean free path of phonons, the energy transport by the phonons is suppressed, causing the value of λ_s_ to decrease. The more complex the microstructure, the longer the heat transfer path through the solid, forming a shielding effect inside, and the smaller the values of λ_s_ and λ_rad_. Low thermal conductivity represents superb thermal insulation performance.

### 2.2. Nanocellulose-Based Thermal Insulative Aerogels

Nanocellulose is mainly divided into cellulose nanocrystals (CNCs), cellulose nanofibrils (CNFs), and bacterial nanocellulose (BNC) [32]. CNCs are short and rod-shaped, with a diameter of about 5–20 nm and a length of about 100–200 nm. CNCs have the characteristics of high crystallinity, strong rigidity, a small ratio of length to diameter, and they does not gel when the concentration is less than 10 wt% [11]. CNFs are filamentous, with a diameter of about 2–20 nm and a length of over 1 µm. CNFs are less crystalline than CNCs and more flexible. Additionally, they can form a physical entanglement network at low concentrations (<1 wt%) [11]. BNC is mainly extracted from bacteria and yeast, which has the defects of a complex preparation process, high energy consumption, and a long production cycle [33]. BNC-based aerogels will not be discussed here. The high crystallinity of CNCs makes CNC-based aerogels easy to deform. In contrast, CNF-based aerogels have excellent flexibility and compressive deformation resistance. Due to the high degree of chain entanglement and high stability, their preparation is relatively easy [11]. Therefore, CNFs are an ideal material for preparing nanocellulose-based aerogels.

For aerogels prepared from the same type of nanocellulose, their thermal insulation performance is primarily influenced by density, pore size, etc. Apostolopoulou-Kalkavoura et al. [34] found that with an increase in CNC-based aerogel density from 40 to 130 kg/m^3^, the porosity decreased from 98.3% to 91.3% and the pore wall thickness increased from 200 nm to 4 μm. Jiménez-Saelices et al. [35] found that the thermal conductivity of a CNF-based aerogel decreased first and then increased with an increase in the density. The solid and radiation conduction of porous thermal insulation materials depends on the density. At higher densities, the contribution of solid conduction increases sharply while the contribution of radiation conduction decreases, inducing an optimum point to minimize the sum of solid phase and radiation conduction contributions. Jiménez-Saelices et al. [36] used spray freeze drying (SFD) to reduce the pores of a CNF-based aerogel from large pores to nanoscale pores. The thermal conductivity decreased from 0.024–0.028 to 0.018–0.021 W m^−1^K^−1^, indicating that reducing the pore size could significantly improve the thermal insulation performance. Previous studies have indicated that enhancing the number of non-uniform particle–particle interfaces can enhance the thermal insulation capabilities of aerogels. Zhou et al. [37] used a step-by-step assembly approach to coat and crosslink CNFs and continuous nanolayers of MOFs, resulting in the preparation of CNF@MOF aerogel. Its thermal conductivity (0.040 W m^−1^K^−1^) was lower than that of the pure CNF aerogel (0.043 W m^−1^K^−1^).

Different types of nanocellulose also lead to different thermal insulation properties of the prepared aerogels. An aerogel prepared from CNFs with a diameter of 3 nm exhibited the lowest thermal conductivity of 0.018 W m^−1^K^−1^, whereas an aerogel prepared from CNCs with a diameter of 10–20 nm showed a thermal conductivity of 0.040 W m^−1^K^−1^ (Table 1) [38,39]. Heidari et al. [40] compared the thermal insulation properties of aerogels composed of CNFs and TEMPO-oxidation pretreated CNFs (TCNFs). The results revealed that TCNFs possessed a finer diameter, with an average size of 5.4 nm, and a completely homogeneous size distribution. In comparison to the CNF aerogel, the TCNF aerogel had thinner pore walls and lower thermal conductivity (CNF aerogel: 35.36 mW m^−1^K^−1^, TCNF aerogel: 33.7 mW m^−1^K^−1^). It could be seen that with an increase in the diameter of the nanocellulose, the characteristic size of the solid phase framework of the aerogel increased, the interfacial thermal resistance was not apparent, and the thermal insulation performance decreased. Zhang et al. [41] found that adding an appropriate amount of CNFs to a CNC aerogel reduced the internal sheet structure, increased the fibril structure, developed the internal network structure, and significantly raised the number of pores and specific surface area. The addition of CNFs reduced the macropores and increased the mesopores, showing more uniform pore distribution and fiber framework. According to Zhu et al. [42], when the suspension concentration was 1.5, 2.5, and 3.5 wt%, the average pore sizes of a CNC-based aerogel were 45, 33, and 30 nm, while those of a CNF-based aerogel were 33, 23, and 23 nm. This indicated that the average pore size of the CNC-based aerogel was larger than the CNF-based aerogel at the same concentration. Yang et al. [43] found that the nanocellulose aerogel had a layered porous structure. CNC aerogel exhibited a relatively dense overall structure with larger pore sizes. As for CNF aerogel, the soft and curved CNFs intertwined and hindered the dense accumulation, and the pore size was small. Compared to the CNC aerogel, the CNF aerogel had more interconnected layered structures. These layered structures demonstrated a relatively uniform pore size, primarily at the nanoscale. The results showed that the thermal conductivity of the CNF aerogel (0.030 W m^−1^K^−1^) was lower than that of the CNC aerogel (0.034 W m^−1^K^−1^). Seantier et al. [44] found that with the addition of the same concentration of CNFs and CNCs to a bleached cellulose fiber aerogel, the thermal conductivity decreased from 0.028 W m^−1^K^−1^ to 0.023 W m^−1^K^−1^ and 0.025 W m^−1^K^−1^, respectively. CNF stiffness is very low compared to that of CNCs. It results in differences not only in terms of interaction towards a BCF but also in terms of increasing entanglement, and, more importantly, in terms of the morphology and porosity of the walls between the BCF formed by the nanofillers. The flexibility and the amorphousness of CNF are likely to have nanometric size pores. In conclusion, CNF is more conducive to improving the thermal insulation performance of the materials. 

## 3. Preparation of Nanocellulose Aerogels

**Figure 3:**
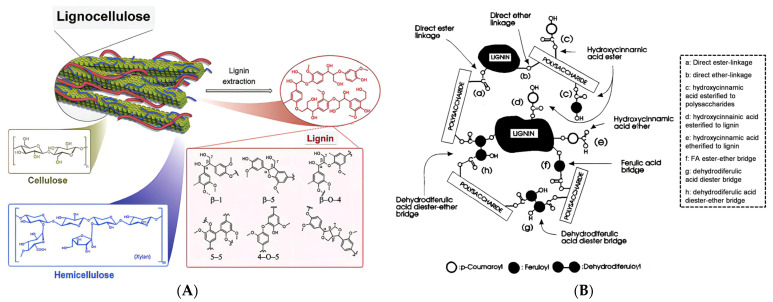
(**A**) Structural schematic of lignocellulose (adapted from [46]). (**B**) Anti-depolymerization barrier in lignocellulose [47].

The process of preparing nanocellulose aerogels from biomass mainly includes cellulose purification, nanocellulose suspension preparation, and drying treatment. For lignocellulosic biomass, the combination of cellulose, hemicellulose, and lignin forms a dense anti-degradation barrier network (Figure 3) [45]. Therefore, it is necessary to remove the lignin and hemicellulose before preparing a nanocellulose suspension. 

### 3.1. Cellulose Purification Method

The methods of cellulose purification are generally classified as physical, biological, and chemical methods. Common physical methods include ultrasound-assisted extraction, steam explosion, mechanical crushing, and others. Singh et al. [48] utilized ultrasound-assisted alkaline urea treatment to extract cellulose from miscanthus, resulting in a cellulose content of 47.8%. Song et al. [49] discovered that steam explosion of kenaf led to efficient degradation of hemicellulose but only a slight degradation of lignin when combined with Fenton oxidation-alkali treatment. The cellulose content was increased to 75.25%. Mechanical crushing is generally used for the activation treatment of fiber raw materials; it can destroy the wrapping structure between cellulose, hemicellulose, and lignin but cannot remove hemicellulose and lignin. The biological method refers to the use of degradative enzymes or microbial strains to degrade hemicellulose and lignin. The method is green, mild, and can retain the cellulose structure well, but the production cycle is long and the extraction efficiency is low [50]. The reaction conditions of enzymatic treatment, such as temperature and pH, must be strictly controlled to guarantee enzymatic activity and avoid affecting the degree of polymerization of nanocellulose caused by enzymatic overreaction [51,52]. Lignin-degrading microorganisms are categorized as white rot fungus, brown rot fungus, soft rot fungus, and others. White rot fungus has the most potent ability to degrade lignin, such as phellius igniarius, funalia gallica, lenzites tricolor, etc. Chemical methods include acid, alkali, and ionic liquid treatments. Among them, acid-base treatment is a relatively mature technology. Melesse et al. [53] treated sugarcane bagasse with sodium hydroxide and obtained 74.71% cellulose, 17.23% hemicellulose, and 9.06% lignin. Yadav et al. [54] used acidic sodium chlorite-sodium hydroxide treatment to extract cellulose from mango wood; the lignin content was reduced to 2% and the hemicellulose content was only 3%. Yang et al. [55] processed balsa wood with choline chloride and acetic acid, and the removal rates of hemicellulose and lignin were 48% and 60%, respectively. He et al. [56] extracted cellulose using acidic sodium chlorite-sodium hydroxide treatment, and they found that the purity of the cellulose obtained by dry crushing the raw material was higher than that obtained by wet beating. The reaction temperature had little effect on the morphology and particle size of the extracted cellulose. However, its crystallinity and thermal stability increased with the reaction temperature.

The physical method results in low pollution and improves the accessibility of reaction reagents to cellulose by breaking down the lignin–carbohydrate complex (LCC). However, pure physical methods, such as steam explosion and mechanical crushing, have a limited effect on cellulose extraction and can destroy its crystal structure, so they are often combined with other methods. The biological method has mild reaction conditions, strong specificity, and results in little environmental pollution, but the effect of using it alone is poor. The chemical method is currently widely utilized for the purification treatment of cellulose due to its high yield and ease of large-scale implementation, and it is evolving towards being green, efficient, cost-effective, pollution-free, and sustainable.

### 3.2. Preparation of Nanocellulose Suspension

Nanocellulose is prepared by mechanical fibrillation of purified cellulose. Mechanical treatments include high-pressure homogenization, microfluidic homogenization, ball milling, ultrafine grinding, high-intensity ultrasonication, pressurized hydrolysis, freeze crushing, steam explosion, and twin-screw extrusion. The principles and characteristics of these methods are shown in Table 2. High-intensity ultrasonication is widely used in nanocellulose preparation. Excessive temperature has a detrimental effect on the crystal structure of nanocellulose, destroying its network and altering the gel behavior of nanocellulose [50]. Hence, ensuring timely cooling during mechanical treatment is crucial to preventing nanocellulose agglomeration due to overheating. 

### 3.3. Drying Method

The high porosity and specific surface area of nanocellulose aerogels make them susceptible to capillary pressure during the drying process, leading to shrinkage, capillary tension, and rupture, ultimately resulting in deformation and collapse of the pore structure [67]. Drying is an important step that affects the structure and properties of nanocellulose aerogels. A complete porous structure favors thermal insulation performance. Frequently used drying methods include atmospheric drying, supercritical drying, and freeze drying.

During atmospheric drying, the surface tension at the gas–liquid interface produces great shrinkage stress, causing the gel matrix to aggregate, collapse, or fragment. Collapse and shrinkage of the pore structure can be decreased by reducing the surface tension of the solvent or strengthening the nano skeleton [68]. Li et al. [68] crosslinked glycidoxypropyltrimethoxysilane (GPTMS) and branched polyethyleneimine (b-PEI) with nanocellulose, after freeze-thawing the mixed suspension and replacing the water in the gel with acetone by soxhlet extraction. An aerogel with a specific surface area of 22.4 m^2^/g was prepared by constant pressure drying at 60 °C. Atmospheric drying is easy to operate, is inexpensive, and has low requirements for equipment. However, it takes a long time, needs a high suspension concentration, and it is challenging to obtain a uniform pore structure [69]. Supercritical drying eliminates the formation of a gas–liquid interface and the generation of capillary pressure during solvent removal, thus maintaining the pore structure [70]. Wang et al. [71] crosslinked CNCs with calcium chloride and prepared a CNC aerogel by supercritical CO_2_ drying (pressure of 120 bar, temperature of 40 °C). The aerogel had a nanoporous network structure composed of mesopores, with a high specific surface area of 353 m^2^/g, an average pore diameter of 8.86 nm, and a minimal micro-shrinkage of only 4.03%. However, supercritical drying needs a large amount of solvent, has a complex preparation process, and is expensive. Freeze drying avoids the influence of surface tension during the gas–liquid phase transition, effectively hinders the occurrence of capillary forces, maintains the skeleton integrity, and prevents the internal network structure from collapsing [72]. However, the nucleation and growth of solvent crystals may disrupt the gel network, forming a macroporous structure [36]. It is necessary to control the parameters such as freezing rate and temperature gradient. Different drying methods lead to different microstructures, resulting in different thermal conductivities. Ebrahimi et al. [69] prepared a nanocellulose aerogel with a thermal conductivity of 0.0417 W m^−1^K^−1^ by atmospheric drying. Yuri Kobayashi et al. [38] produced a nanocellulose aerogel by supercritical drying, and the lowest thermal conductivity was 0.018 W m^−1^K^−1^. Jiménez-Saelices et al. [73] used freeze drying to obtain a nanocellulose aerogel with a thermal conductivity as low as 0.024 W m^−1^K^−1^. At present, freeze drying is mainly used to prepare nanocellulose aerogel.

## 4. Mechanical Property Improvement of Nanocellulose Aerogels

The mechanical properties of nanocellulose aerogels are an important factor restricting their application. There have been a lot of studies on improving the mechanical properties of nanocellulose aerogels, mainly focused on compression and elasticity. The regulation of morphology and structure, chemical crosslinking, and introduction of enhanced components are frequently employed to increase the mechanical strength.

### 4.1. Regulation of Morphology and Structure

The concentration of the nanocellulose suspension and the drying method are the main factors affecting aerogel structure, and the structure restricts the mechanical properties [74,75].

#### 4.1.1. Changing the Suspension Concentration

**Figure 4:**
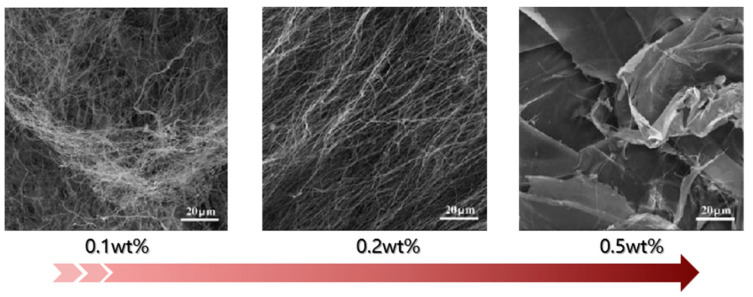
SEM images of nanocellulose aerogels with different suspension concentrations (adapted from [76]).

The density of nanocellulose aerogels increases with the concentration of the nanocellulose suspension. Thus, the microstructure can be controlled by changing the suspension concentration. As shown in Figure 4, at a low concentration, the nanocellulose aerogel is composed of high-aspect-ratio nanofibers and presents a three-dimensional network entanglement structure. When the concentration is gradually increased, dense two-dimensional sheet-like structures are formed, organized by fine nanofibers [76].

Zhang et al. [77] found that the compressive strength, yield strength, and Young’s modulus of a nanocellulose aerogel increased with the suspension concentration. On the one hand, the density of the aerogel increased with the nanocellulose content; on the other hand, the higher the nanocellulose content, the more two-dimensional sheet-like structures are formed, which could stabilize the initial shape of the aerogel under the action of external force. Gupta et al. [78] found that when the suspension concentration was 0.75–1 wt%, the aerogel exhibited plastic deformation under compressive strain. However, at a suspension concentration of 1.25–1.75 wt%, the aerogel showed apparent yielding under compression strain, indicating that the increase in suspension concentration improved the load-carrying capacity. It is worth noting that with the increase in the concentration of the nanocellulose suspension, the density of the nanocellulose aerogel increases gradually while the specific surface area decreases continuously. This reduces the air volume captured by the pores inside the aerogel and increases its solid conduction, increasing the total thermal conductivity and affecting its thermal insulation performance.

#### 4.1.2. Regulation of Freeze Drying Parameters

Since freeze drying is mainly used to prepare nanocellulose aerogels, this review primarily discusses the impacts of freeze drying parameters. The precursor dispersion needs to be pre-frozen before freeze drying. This step is divided into cryogenic freezing and ultra-low temperature freezing according to the freezing temperature. Based on the temperature gradient, this can be categorized into directional and random freezing. The ice crystal structure can be controlled by adjusting the freezing conditions, thereby regulating the internal network structure of the aerogel.

**Figure 5:**
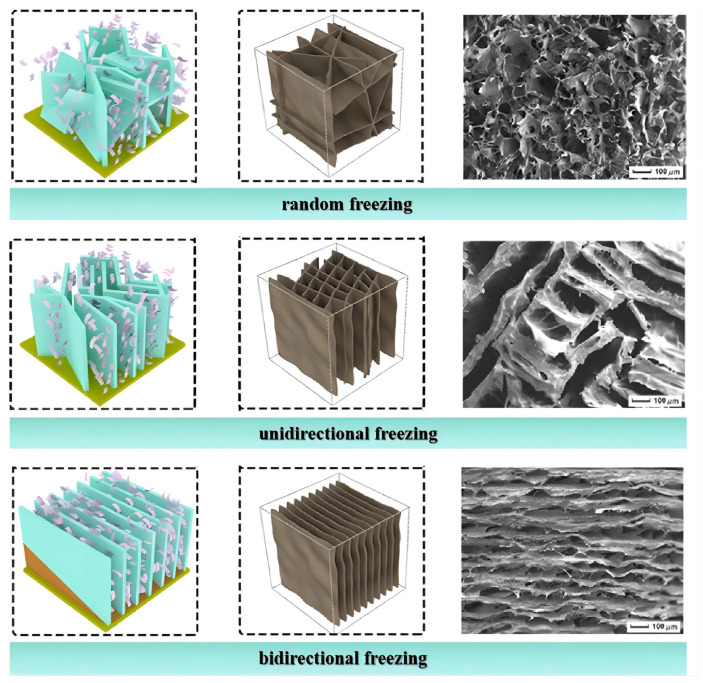
Microstructure of nanocellulose aerogels prepared using different freezing methods (adapted from [88]).

When the cryogenic freezing temperature is relatively high, a slower freezing rate enhances the formation of larger pore structures. Ultra-low temperature freezing makes the ice crystals grow rapidly, leaving dense and tiny pores in the aerogel after sublimation. For instance, a CNF/PVA aerogel prepared by freezing at −196 °C had a pore size of 20 µm. When the temperature was increased to −26 °C, the pore size increased to 200 µm [79]. Figure 5 shows the formation of microstructures of nanocellulose aerogels is influenced by temperature gradients. Directional freezing means applying a directed temperature gradient to the precursor dispersion before freeze drying so that the solvent ice crystals grow along the freezing gradient direction on the cold source surface [80]. Directional freezing mainly includes unidirectional and bidirectional freezing [81]. Unidirectional freezing refers to freezing applied only at the bottom of the container, with the precursor dispersion in direct contact with the cold source, forming a temperature gradient along the vertical direction and forcing ice crystals to grow from the bottom to the top [82]. Covering the cold source with a polydimethylsiloxane (PDMS) wedge forms temperature gradients in both the horizontal and vertical directions, controlling the nucleation and growth of ice crystals [83,84]. Random freezing refers to freezing without applying a directed ice template to the precursor dispersion. In subfreezing environments, ice crystals nucleate randomly in various orientations through slow heat exchange between the liquid and gas [85]. Random freezing can produce an isotropic aerogel, and its microporous structure is disordered [86]. An anisotropic aerogel can be obtained by directional freezing. Unidirectional freezing forms an ordered honeycomb structure [87]. Bidirectional freezing forms a parallel lamellar architecture with numerous multi-arch microstructures and interconnected pores through different layers [88].

Liang et al. [89] compared the mechanical properties of aerogels prepared by random and directional freezing. The results showed that directional freezing bestowed the aerogel with a higher compressive modulus and compressive stress. The aerogel prepared by bidirectional freezing exhibited good elasticity (85% stress retention after 10 cycles) and high compressibility (compressive stress up to 340 kPa at 90% strain) [85]. Recently, Qin et al. [90] developed a new dual ice-templating assembly (DITA) strategy to prepare nanocellulose aerogel. It imparted excellent isotropic elasticity (as low as 8.2% unrecoverable strain after 50 cycles) and the aerogel demonstrated superelasticity at an extremely low temperature. Directional freezing efficiently regulates the morphology and structure of nanocellulose aerogels and forms a regular microstructure to significantly improve the mechanical properties.

### 4.2. Chemical Crosslinking

**Figure 6:**
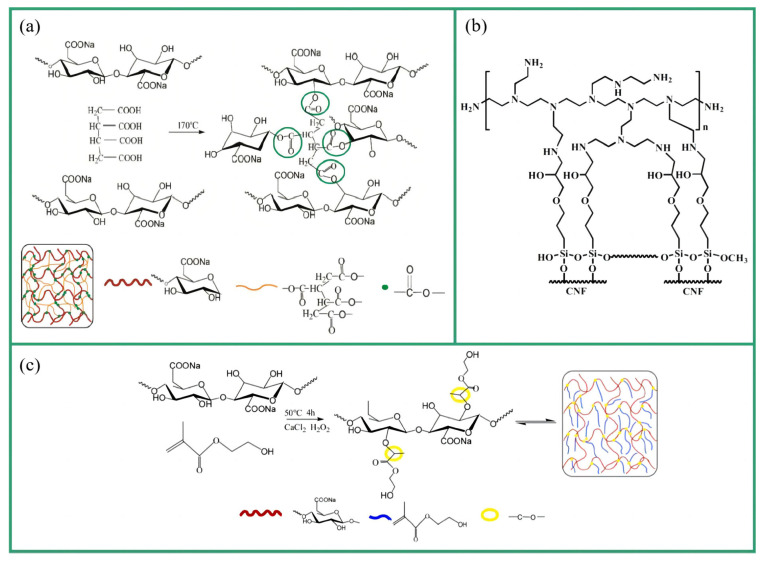
Crosslinking mechanisms: (**a**) the esterification of 1,2,3,4-butanetetracarboxylic (BTCA) and TEMPO-mediated oxidation cellulose nanofibrils (TCNFs) [95]; (**b**) the etherification of γ-glycidoxypropyltrimethoxysilane (GPTMS), branched polyethylenimine (PEI), and cellulose nanofibers (CNFs) [98]; (**c**) grafting 2-hydroxyethyl methacrylate (HEMA) onto CNFs via radical polymerization [97].

Nanocellulose aerogels are formed by physical or chemical crosslinking between molecular chains [91]. Crosslinking can not only form a three-dimensional network structure but also improves the mechanical properties. The network structure of pure nanocellulose aerogels is mainly formed by hydrogen bonds on their surface and entanglement between nanocellulose molecules. These interactions are reversible, resulting in an unstable structure susceptible to chemical environment changes and mechanical forces [92]. Chemical crosslinking forms irreversible covalent bonds inside aerogels by introducing a crosslinker and strengthening the crosslinking points of the three-dimensional network [93]. The crosslinker impedes the relative sliding of the nanocellulose molecular chain, avoids the generation of random pores, and contributes to forming a complete lamellar structure. Selecting an appropriate crosslinker is an effective method to improve the mechanical properties of nanocellulose aerogels. Figure 6 shows several crosslinking mechanisms.

Jiang et al. [94] crosslinked cellulose nanofibers (CNF) with methylene diphenyl diisocyanate (MDI) to form urethane bonds. The aerogel retained a honeycomb structure, showing improved mechanical properties; with an increase in the MDI concentration, Young’s modulus increased to 209 kPa, the yield strength increased to 13.8 kPa, and the ultimate strength increased to 66 kPa, which were nearly two-, four-, and one-time increases compared with the uncrosslinked aerogel. Chen et al. [95] used 1,2,3,4-butanetetracarboxylic acid (BTCA) as a crosslinker to form a crosslinked network with TCNFs via esterification. The process involved pre-freezing the mixture at −25 °C for 12 h, freeze drying for 48 h, and heating at 170 °C for 3 min. As a result, an aerogel with excellent flexibility and enhanced elasticity (elasticity increased by 76.3% at 70% strain) was successfully synthesized. Chen et al. [96] used blocked waterborne polyurethane (BWPU) to crosslink with CNFs. The mixture was freeze-dried for 72 h after directional freezing in liquid nitrogen and then heated at 160 °C for 10 min to obtain the final sample. The aerogel regained its initial height under 20%, 40%, and 60% compressive strain, and the stress–strain curve showed good recovery. Moreover, it maintained good thermal insulation performance even after 60% compression strain. Chen et al. [97] used 2-hydroxyethyl methacrylate (HEMA) as the crosslinker and calcium chloride (CaCl_2_) as the initiator to form a crosslinked network with TCNFs through radical polymerization, which was then freeze-dried for 48 h after directional freezing in liquid nitrogen to prepare an aerogel. The aerogel exhibited excellent compressibility and shape recovery, with a maximum unrecoverable strain of only 6.5% at 70% strain, and the storage modulus was as low as 20 Pa. Cheng et al. [98] crosslinked CNFs with γ-glycidoxypropyltrimethoxysilane (GPTMS) and branched polyethylenimine (PEI) via etherification. The mixture was freeze-dried at −50 °C for 24 h and then heated at 110 °C for 30 min. After washing with deionized water and acetone, it was vacuum-dried at 30 °C for 24 h to prepare a robust and flexible aerogel (the shape recovery rate was up to 93% from 50% compression strain).

### 4.3. Introduction of Enhanced Components

The enhanced components introduced to improve the mechanical properties of nanocellulose aerogels include inorganic nanocomponents and resilient organic materials [95]. Precursor mixing and direct impregnation are popular methods to introduce enhanced components. Precursor mixing denotes adding enhanced components into the nanocellulose suspension to strengthen the aerogel network skeleton. Direct impregnation immerses the nanocellulose aerogel in a solution containing enhanced components to form an interpenetrating network.

Chen et al. [99] immersed a pure CNF aerogel in a hydrolysate of methyltriethoxysilane (MTES) and placed it in an alkaline solution (pH 10). Following two-step impregnation, the sample was placed in anhydrous ethanol for solvent replacement for 12 h. Finally, it was vacuum-dried at 80 °C for 5 h. After introducing polysiloxane particles into a nanocellulose aerogel, the density was increased from 0.0592 to 0.0950 g/cm^3^ and the compression modulus was enhanced from 441.43 to 1572.87 KPa. Liu et al. [100] incorporated boron nitride nanosheets (BNNSs) into a CNF suspension and freeze-dried the mixture for 48 h after being frozen at −25 °C for 12 h to form an aerogel. The addition of BNNSs led to a rough fiber framework, and the strong interaction between CNFs and BNNSs formed an aerogel with a three-dimensional porous structure and a dense wall. Introducing BNNSs increased the density from 0.03 to 0.062 g/cm^3^, improved the compressive strength from 0.25 to 0.37 MPa, and enhanced the compressive modulus. Liu et al. [101] obtained an aerogel with excellent compressibility and resilience by adding carbon nanotubes (CNTs) and graphene oxide (GO) to the nanocellulose suspension. The aerogel exhibited a regular honeycomb structure when the CNF/CNT/RGO mass ratio was 4:2:3, which could avoid sliding and splitting perpendicular to the compression direction and was more conducive to storing elastic energy. It endured 80% strain without obvious deformation and maintained a stress retention rate of 83.8% after 100 compression cycles. It even withstood 10,000 compression cycles, with stress retention rates of 86.1% at 30% strain and 85.3% at 50% strain. Introducing enhanced components can improve the mechanical properties of nanocellulose aerogels, but it often sacrifices the ultra-light feature, negatively affects flexibility, and causes two-phase/polyphase interface bonding problems.

### 4.4. Summary

Adjusting the morphology of nanocellulose aerogels can optimize their internal structure, improve their bearing capacity, and better maintain their initial shape under external force. Nevertheless, only restructuring the morphology cannot significantly enhance their compression resilience. Chemical crosslinking forms irreversible covalent bonds between nanocellulose molecules, enhancing their resistance to sliding during deformation, which is an effective way to improve the compression resilience of nanocellulose aerogels. The addition of enhanced components can improve the strength of the skeleton. However, it is not as effective as chemical crosslinking in improving the compression resilience. Table 3 summarizes the modifying effects and limitations of different methods.

## 5. Flame Retardancy Modification of Nanocellulose Aerogels

Introducing flame retardants into nanocellulose aerogels can effectively improve their flame retardancy. Flame retardants are mainly inorganic, organic, and organic/inorganic composite flame retardants.

### 5.1. Inorganic Flame Retardants

Inorganic flame retardants have the advantages of low toxicity, environmental friendliness, and low price, and they are widely used in flame retardant areas. At present, inorganic flame retardants for nanocellulose aerogels mainly include metal hydroxide, metal oxide, montmorillonite, phosphorus-based flame retardant (such as ammonium polyphosphate), carbon-based flame retardant (such as GO), and others [102,103,104,105]. Luo et al. [106] incorporated MgAl-layered double hydroxide to prepare two series of aerogels. Compared to pure nanocellulose aerogel, the peak heat release rate (pHRR) was reduced by 41% and 50% and the smoke production ratio (SPR) was reduced 79% and 75%, respectively. Yang et al. [107] added molybdenum disulfide (MOS_2_) to form a carbon protective layer on the aerogel, increasing the pyrolysis temperature to 300–400 °C, reducing the mass loss rate to 60%, and improving the limiting oxygen index (LOI) to 34.7%. The composite aerogel also showed excellent self-extinguishing characteristics. Gupta et al. [108] found that after doping with sepiolite clay, the aerogel was extinguished after 0.5 s of combustion. Huang et al. [109] introduced microencapsulated ammonium polyphosphate (MCAPP) into an aerogel to increase the residual carbon after burning. Wan et al. [110] added GO to an aerogel; it maintained its shape and self-extinguished instantaneously without smoke released during burning. However, inorganic flame retardants often need a large addition or combination to achieve an excellent flame retardant effect, which will destroy the three-dimensional network structure of the aerogel and affect its mechanical and thermal insulation properties to a certain extent.

### 5.2. Organic Flame Retardants

Compared with inorganic flame retardants, organic flame retardants have good compatibility with nanocellulose aerogels, high flame retardant efficiency, and low addition. Organic flame retardants containing phosphorus and nitrogen are the most widely studied. Guo et al. [111] added N-methylol dimethylphosphonopropionamide (MDPA) to an aerogel via crosslinking reactions; it self-extinguished, had a high residual carbon rate, and the LOI value increased obviously with the addition of MDPA. In addition, the aerogel exhibited perfect flexibility, resilience, and thermal insulation. Jiang et al. [94] used methylene diphenyl diisocyanate (MDI) to improve the thermal stability of an aerogel, which reduced its thermal decomposition rate and increased the residual carbon rate to 43% at 500 °C. The crosslinking of MDI and CNFs endowed the aerogel with good mechanical properties as well. Ren et al. [112] modified an nanocellulose aerogel by in situ supramolecular assembly of melamine (MEL) and phytic acid (PA). It self-extinguished and kept its intact shape after being ignited. The weight losses were as low as 12.2% and 13.6% in the horizontal and vertical burning tests. Moreover, the mechanical properties were enhanced and good thermal insulation was maintained. However, organic flame retardants produce toxic gas during combustion, causing damage to human health and the environment.

### 5.3. Organic/Inorganic Composite Flame Retardants

Composite flame retardants combine the advantages of inorganic and organic flame retardants. Yue et al. [113] used the synergistic effect between sodium alginate (SA), boric acid (BA), and CaCO_3_ to improve the flame retardancy of a nanocellulose aerogel. Its LOI value was up to 35.6%, the vertical combustion test (UL-94) rating reached the V-0 level, and the pHRR and total heat release (THR) decreased to 27.4 W/g and 3.7 kJ/g, respectively. Furthermore, BA crosslinked with SA and CNFs to form borate ester bonds, resulting in a more compact pore structure in the aerogel, which led to the compressive modulus and specific modulus increasing to 22.8 kPa and 2781.7 kPa g^−1^m^−3^, respectively. The crosslinking of Ca^2+^ and SA formed an ‘eggbox’ structure with an interpenetrating network and strengthened the intermolecular interaction in the aerogel, which increased the compressive modulus and specific modulus to 28.40 kPa and 3890.41 kPa g^−1^m^−3^, respectively. Under the combined effects of chemical and ionic crosslinking, the uniformity of the aerogel pore size was improved, increasing the compressive modulus and specific modulus to 28.67 kPa and 2955.67 kPa g^−1^m^−3^, respectively. The results showed that the composite flame retardant could reduce the pore size and porosity of the aerogel through crosslinking, and the increase in crosslinking degree strengthened the framework structure, thus improving its mechanical properties. Köklükaya et al. [114] deposited a flame retardant coating composed of cationic chitosan (Ch), anionic poly (vinylphosphonic acid) (PVPA), and anionic montmorillonite clay (MMT) on a nanocellulose aerogel using layer-by-layer (LbL) fabrication technology. The LbL coating evenly covered the surface of the aerogel without altering the pore structure. It immediately extinguished the flame and eliminated flameless combustion (afterglow), which helped retain the original shape of the aerogel during combustion. The LbL-treated aerogel showed a low pHRR of 31 kW/m^2^. Guo et al. [115] utilized pentaerythritol phosphate melamine salt (PPMS) in combination with a melamine-urea-formaldehyde (MUF) pre-polymer to improve the flame retardancy. The synergistic effect between MUF and PPMS endowed the aerogel with excellent self-extinguishing characteristics. Its char residue at 600 °C and LOI value rose to 38.8% and 30.2%, respectively, while the pHRR, heat release capacity (HRC), and temperature of the pHRR were reduced to 37.6 W/g, 26 J/(g·K), and 309 °C, respectively. The introduction of the composite flame retardant did not negatively impact the thermal insulation of the aerogel, and its thermal conductivity was maintained at approximately 30 mW/(m·K). However, there are some problems in using composite flame retardants, such as poor compatibility and complex preparation processes.

### 5.4. Summary

Inorganic flame retardants can promote the carbonization of materials during combustion. They act as a barrier, slowing combustible gas diffusion and inhibiting the shrinkage of nanocellulose aerogels. The flame retardancy of nanocellulose aerogels increases with the addition of inorganic flame retardants. However, introducing them leads to a decrease in thermal insulation and mechanical properties. It is necessary to improve and optimize the comprehensive performance of nanocellulose aerogels. The cellulose surface contains numerous hydroxyl groups that enable the introduction of organic flame retardants through crosslinking reactions. The crosslinked structure makes nanocellulose aerogels hard to ignite on both micro and macro scales. Organic flame retardants induce higher efficiency in flame retardation and can enhance the mechanical properties of nanocellulose aerogels to some degree compared to inorganic flame retardants. Nevertheless, the pyrolysis process of organic flame retardants generates toxic and harmful gases. The synergistic effect of composite flame retardants contributes to forming a protective carbon layer, enhancing the free radical capture ability and gas barrier effect. In contrast, there exist some problems, such as complicated preparation processes and poor compatibility. Table 4 summarizes some methods of modifying flame retardancy.

## 6. Hydrophobicity Modification of Nanocellulose Aerogels

**Figure 7:**
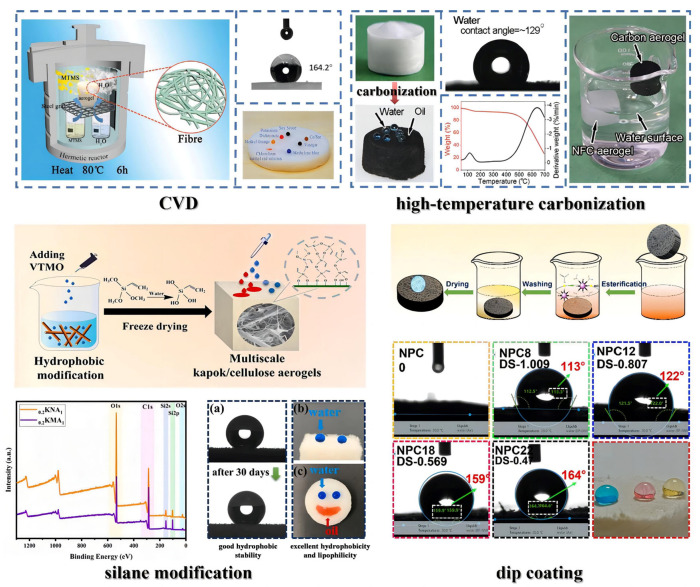
Common hydrophobic modification methods: CVD (adapted from [90]), high-temperature carbonization (adapted from [120]), silane modification (adapted from [121]), and dip coating (adapted from [122]).

Current hydrophobic modification methods of nanocellulose aerogels mainly include chemical vapor deposition (CVD), dip coating, silane modification, high-temperature carbonization, and others (Figure 7).

CVD uses a volatile organosilane as a modifier to deposit on the surface of nanocellulose, combining with its hydroxyl groups to form a polysiloxane hydrophobic coating. The commonly used reagents for CVD are primarily organosilanes, such as methyltrichlorosilane (MTCS), methyltrimethoxysilane (MTMS), octyltrichlorosilane (OTS), and so on [123,124,125]. Rafieian et al. [126] modified a nanocellulose aerogel using CVD with hexadecyltrimethoxylan (HDTMS). The modified aerogel had a maximum water contact angle (WCA) of 139° and kept a low density (11–17.5 mg/cm^3^) and high porosity (98.8–99.3%). Guan et al. [127] modified an aerogel with MTMS via CVD. Its WCA was up to 151° and it maintained a particular springy-like lamellar structure as before modification. The modified aerogel exhibited a compressive strain resistance of 60% and recovered to its original height after stress release. Furthermore, even after 100 compression cycles at 40% strain, it still recovered up to 93% of its initial height. These results demonstrated that the modified aerogel had excellent compression resilience and fatigue resistance. CVD is simple to operate and can maintain the pore structure to the utmost extent. However, it is challenging to uniformly deposit hydrophobic coatings inside the three-dimensional porous aerogel using CVD.

The dip coating technique coats hydrophobic materials onto nanocellulose aerogels through intermolecular interactions, which has the advantage of a simple preparation process. The dip solution is usually prepared by mixing organic solvents (ethanol, acetone, toluene, etc.) and low surface energy materials (silane, fatty acids, etc.). Chatterjee et al. [128] coated graphene nanosheets on a nanocellulose aerogel, reaching a WCA of 130°. The graphene nanosheets attached to the pore surfaces to form a uniform and dense monolayer without compromising the pore size distribution, porosity, and elasticity. Chhajed et al. [129] chose stearic acid chloride (SAC) as a dip coating material to modify the surface of a nanocellulose aerogel by esterification reaction dip coating. Due to the substitution of nonpolar aliphatic chains for hydroxyl groups on the surface and the decrease in polar components, the surface roughness of the modified aerogel was significantly increased, leading to a WCA of 159°. However, the pore size of the cellular structure was not affected (the pore size was in the range of 3–20 nm before and after modification). Dip coating can eliminate solvent effects in the nanocellulose aerogel preparation process. However, attention must be paid to avoid collapsing the pores and reducing the surface area during drying.

Silane modification refers to adding polysiloxane sol in the nanocellulose suspension and forming a polysiloxane layer on the nanocellulose surface via dehydration and self-condensation to obtain hydrophobicity. Liu et al. [130] successfully grafted modifying monomers onto cellulose nanofibers by adding perfluorohexyl ethyl trimethoxysilane (PFOTMS) and 3-aminopropyl trimethoxysilane (KH540) to the suspension. Finally, a superhydrophobic aerogel was obtained (WCA was 151°) after freeze drying. The modified aerogel formed a regularly arranged and more compact three-dimensional network structure. Vinyltrimethoxysilane (VTMO) was added to a nanocellulose suspension to obtain a modified aerogel with good formability and structural stability after freeze drying. Its WCA was as high as 147.6° [121]. Silane modification can not only modify the hydrophobicity of nanocellulose aerogels but also improve their porosity, mechanical properties, and thermal insulation performance. However, the hydrophobic modification of nanocellulose before preparing a nanocellulose aerogel may affect its subsequent synthesis.

Cellulose has a high carbon content; carbonizing nanocellulose aerogels at high temperatures can prepare hydrophobic carbon aerogels. Zhou et al. [131] heated an aerogel in a tube furnace to 500 °C at a heating rate of 5 °C/min under argon protection, which was maintained for 120 min. After naturally cooling to room temperature, a hydrophobic carbon aerogel was obtained. Its WCA increased to 140°, with low density (6.17 mg/cm^3^), high porosity (99.61%), and good flexibility (returned to 95% of the original height even after 15 compression cycles at 50% strain). Chen et al. [120] carried out similar high-temperature carbonization on a nanocellulose aerogel and obtained a hydrophobic carbon aerogel (WCA was up to 129°) with a low density (7.8 mg/cm^3^), high resilience and flexibility (withstood 70% compressive strain and almost recovered to the original volume after stress release; the compressive stress at 70% strain was 9.9 kPa), and high thermal stability (pyrolysis temperature > 400 °C). However, high-temperature carbonization may destroy the internal pore structure of the aerogel and usually requires high energy consumption.

CVD can maintain the pore structure of nanocellulose aerogels to the greatest extent without negatively affecting their mechanical properties and thermal insulation performance. However, the uneven graft distribution of the modifier within the material makes the hydrophobic layer easily destroyed under the action of external force. Dip coating enhances the uniformity of hydrophobicity, but it can cause pores to collapse during the subsequent drying process, affecting the mechanical and thermal insulation properties. Silane modification improves the comprehensive properties of nanocellulose aerogels while achieving hydrophobic uniformity, yet it may impact the subsequent synthesis and functional utilization. High-temperature carbonization has high energy consumption and a complicated manufacturing process, and if the carbonization temperature is too high, the pore structure of the nanocellulose aerogel will collapse. The attainment of uniform hydrophobicity in nanocellulose aerogels while preserving their formability and internal structure is worthy of further study.

## 7. Conclusions and Outlook

As a third-generation aerogel after inorganic and polymer aerogels, nanocellulose aerogels have both the functional characteristics of a traditional aerogel and a wide range of other characteristics, such as sources, renewability, good biocompatibility, degradability, and others. In addition, the surface of nanocellulose is rich in hydroxyl groups, which enables the function to be easily adjusted, and it is expected to be used as a high-performance thermal insulation material.

(I) The heat transfer mechanism of nanocellulose aerogels includes gas conduction, solid conduction, and radiation. The thermal insulation performance of nanocellulose aerogels is influenced by the size effect, interface effect, and pore size distribution. The thermal insulation performance of nanocellulose aerogels can be improved as follows: (i) reducing the pore size below the mean free path of air; (ii) increasing the uniformity of pore size distribution; (iii) lessening the characteristic size of the solid phase framework; (iv) choosing the appropriate density; and (v) building a multi-level ordered microstructure. In considering the coupling effect, the increase in porosity decreases the coupled thermal conductivity, according to the formula. Theoretically, increasing the porosity while controlling the pore size can improve the thermal insulation performance of nanocellulose aerogels. However, there is still a lack of research on the porosity and thermal insulation performance of nanocellulose aerogels.
(II) The yield of cellulose purified by chemical methods is high, making it suitable for large-scale processing. Using high-intensity ultrasonication to fibrillate purified cellulose is easy to operate and can obtain nanocellulose with a high aspect ratio. Freeze drying maintains the skeleton integrity, prevents the internal network structure from collapsing, and is easy to implement. Therefore, the ideal method to prepare nanocellulose aerogels from biomass materials is as follows: (i) removing the lignin and hemicellulose by chemical methods to obtain purified cellulose; (ii) preparation of nanocellulose suspension using high-intensity ultrasonication to fibrillate the purified cellulose; and (iii) freeze dry the suspension to obtain the nanocellulose aerogel.
(III) Increasing the concentration of the nanocellulose suspension results in forming more two-dimensional sheet structures in the aerogel, stabilizing its initial shape under the action of external force. During the freezing casting, the growth rate of ice crystals increases with the decrease in temperature, resulting in the formation of small pore sizes. Additionally, directional freezing causes ice crystals to grow along the freezing gradient direction, forming an ordered structure inside the aerogel and enhancing its structural stability. Chemical crosslinking forms irreversible covalent bonds between nanocellulose molecules, enhancing their resistance to sliding during deformation. The introduction of enhanced components can improve the strength of the skeleton, leading to an increase in its compressive strength. However, increasing the suspension concentration will increase the density of the nanocellulose aerogel and affect its thermal insulation performance. Introducing enhanced components increases the aerogel density, negatively affects the flexibility, and causes interface bonding problems. It is challenging to meet practical application requirements only by adjusting the freeze drying parameters. Chemical crosslinking is an effective method for improving the compression resilience of nanocellulose aerogels. For instance, the shape recovery rate could reach 93% under 50% compression strain.
(IV) The flame retardants for nanocellulose aerogel include inorganic, organic, and composite flame retardants. Inorganic flame retardants are green and cheap but often need a large addition to achieve an excellent flame retardant effect (e.g., adding 10.95 wt% molybdenum disulfide to an aerogel achieved good flame retardancy). Organic flame retardants have good compatibility with nanocellulose aerogels and require a low addition (e.g., adding 1.0 wt% melamine and phytic acid bestowed a nanocellulose aerogel with good flame retardancy), but they produce toxic gas during combustion. Composite flame retardants combine the advantages of inorganic and organic flame retardants. However, they have some problems, such as poor compatibility and complex preparation processes. Developing flame retardants with efficient flame retardancy, good compatibility, and environmentally friendliness is urgently needed. Modifying flame retardants by the use of surfactants, microencapsulation, grafting reactions, plasma/UV treatment, and other methods can enhance their flame retardant efficiency, compatibility with substrates, and durability.
(V) The existing hydrophobic modification processes for nanocellulose aerogels are usually complex and energy intensive. As the most widely used strategy, CVD has the disadvantage of uneven hydrophobic modification. Due to the fragile skeleton structure of nanocellulose aerogels, the dip coating technique may cause pore collapse during drying. Silane modification can achieve hydrophobic uniformity of nanocellulose aerogels and improve their porosity and mechanical properties, but it is difficult to accomplish in large-scale applications. High-temperature carbonization has high energy consumption and a complicated manufacturing process, and if the carbonization temperature is too high, the nanocellulose aerogel will be crushed. The following should be considered in future work: (i) introducing soft elastic materials with good compatibility to mitigate the negative impact of external energy on the structure and stability of nanocellulose aerogels through elastic deformation; (ii) adding nano self-healing materials to refine the durability and mechanical properties of hydrophobic nanocellulose aerogels; and (iii) developing green, non-toxic, and recyclable modifiers.

## Figures and Tables

**Table 1:** Thermal conductivities of some nanocellulose aerogels.

Drying Method	Nanocellulose Type	Nanocellulose Diameter/nm	Other Components	Pore Size	Thermal Conductivity/mW m^−1^K^−1^	Refs.
Supercritical drying	CNF	3	-	30 nm	18–38	[38]
Supercritical drying	CNC	10–20	-	10 nm–1 µm	40–75	[39]
Freeze drying	CNF	5.7, 16.1	-	-	35.36	[40]
Freeze drying	TCNF	5.4	-	-	33.7	[40]
Freeze drying	CNF	-	-	-	24–28	[36]
Spray freeze drying	CNF	-	-	10–100 nm	18–21	[36]
Freeze drying	CNF	20	MOF	-	41	[37]
Freeze drying	CNF	20 ± 4	Bleached cellulose fibers	5 nm	23	[44]
Freeze drying	CNC	7 ± 2	Bleached cellulose fibers	6–7 nm	25	[44]

**Table 2:** The principles and characteristics of different mechanical treatments.

Mechanical Treatment	Principle	Characteristics	Refs.
High-pressure homogenization	The energy generated by high pressure and high-speed movement breaks down the compact structure of fibers, resulting in a reduction in fiber size.	The machine is easily blocked, so it is necessary to control the suspension concentration and fiber size.	[57]
Microfluidic homogenization	The booster pump pumps the suspension into the small Z-shaped pipeline under high pressure, and fiber opening occurs under the high shear force and high-speed impact force.	(1) Many cycles; (2) High energy consumption; (3) Significant negative impact on the environment; (4) Challenging to realize large-scale application.	[58,59]
Ball milling	A steel or stone ball is utilized in the ball mill to break and impact the fiber.	This method destroys the crystalline domains of cellulose, leading to a decrease in nanocellulose crystallinity, thus affecting its mechanical and thermal properties.	[60]
Ultrafine grinding	The strong shear force generated by the rotation of the grindstone destroys the cell wall and hydrogen bonding of fibers.	(1) The machine has a simple structure and stable operation; (2) It can handle highly concentrated suspensions; (3) No need to control fiber size; (4) Grinding stone friction produces a stone powder that contaminates the slurry and is hard to separate efficiently.	[61]
High-intensity ultrasonication	The cavitation effect caused by high-intensity ultrasound destroys the hydrogen bonds between fiber chains and decomposes cellulose.	(1) Easy to operate; (2) No limitation on the concentration of the sample, and it is possible to prepare a high-concentration nanocellulose suspension; (3) Easy to observe the sample; (4) Low shear force is applied to cellulose, preserving natural fiber length.	[62]
Pressurized hydrolysis	Subcritical water is used to hydrolyze cellulose with its high ionization efficiency, activity, and diffusion.	(1) Requires high temperature and pressure; (2) High energy consumption.	[63]
Freeze crushing	The compression of ice crystals breaks the cell wall structure and obtains nanocellulose under high shear force.	It is often used in combination with other treatments.	[64]
Steam explosion	Use of the explosive power of high-pressure steam in a short time to destroy the fiber structure.	The energy consumption is relatively low.	[50]
Twin-screw extrusion	During the extrusion process, the shear force coming from screw rotation disintegrates the fibers.	(1) Can produce nanocellulose at a high solids content; (2) Lower energy consumption	[65,66]

**Table 3:** Effects and limitations of different modification methods.

Methods	Mechanical Properties	Limitations	Refs.
Increase the suspension concentration	A compressive strength of 0.227 MPa, Young’s modulus of 0.223 MPa, and yield stress of 27.47 kPa	(1) Increases the density; (2) Reduces the thermal insulation performance.	[77]
Regulation of freeze drying parameters	The compressive stress at 90% strain reached 340 kPa and exhibited 85% stress retention after 10 cycles.	Improving the mechanical properties of aerogels solely through adjustments in freeze drying parameters cannot meet the requirements of practical applications.	[85]
Chemical crosslinking	The maximal shape recovery from a 50% compression strain was up to 93% of its original thickness.	The current crosslinking strategy has defects, such as complex crosslinking processes or the use of toxic crosslinking agents.	[97]
Introduction of enhanced components	Withstood a high strain of 80% without remarkable geometric deformation and displayed high stress retention of 83.8% after 100 cycles	(1) Increases the density; (2) Affects the flexibility; (3) Resulst in interface bonding problems.	[101]

**Table 4:** Some flame retardant modification methods.

Methods	Advantages	Limitations	Refs.
Surfactant method	(1) Improves the compatibility and storage stability of flame retardants; (2) Reduces the impact of flame retardants on material properties.	The effect of nonionic surfactant modification on inhibiting combustion and reducing smoke release is not obvious.	[116]
Microencapsulation	(1) Improves flame retardant efficiency; (2) Enhances the mechanical properties and hydrophobicity of materials.	increase the quality of flame retardants.	[117]
Grafting reaction	(1) Improves the dispersion of flame retardants in the matrix; (2) Enhances flame retardant efficiency; (3) Reduces the impact on material mechanical properties.	Complex preparation processes.	[118]
Plasma/UV treatments	(1) Strengthens the combination of flame retardant and substrate; (2) Improves the durability of flame retardants.	Inapparent improvement of flame resistance.	[119]

## Data Availability

Not applicable.
